# Perceived Discrimination and Psychological Well-Being Among Immigrants Living in Greece: Separation as Mediator and Interdependence as Moderator

**DOI:** 10.5964/ejop.1531

**Published:** 2022-02-25

**Authors:** Evangelia Kateri, Donna Papastylianou, Evangelos Karademas

**Affiliations:** 1 Department of Psychology, University of Crete, Rethymno, Greece; 2 Department of Psychology, Νational and Kapodistrian University of Athens, Athens, Greece; University of Liverpool, Liverpool, United Kingdom

**Keywords:** acculturation attitudes, perceived discrimination, self-construal, psychological well-being, immigrants

## Abstract

The present study was based on the rejection-identification model regarding migrants acculturation. Personal perceived discrimination, acculturation attitudes, self-construal, and psychological well-being were examined simultaneously in Albanian and Indian immigrants residing in Greece (N = 233). It was hypothesized that perceived discrimination would be related negatively to immigrants’ psychological well-being, both directly and indirectly. A positive relationship was expected between perceived discrimination and separation and a negative relationship between discrimination and integration, or assimilation. It was, also, expected a positive relationship of perceived discrimination to interdependent self-construal and a negative relationship to independent self-construal. Furthermore, it was examined the mediating role of separation in the association of perceived discrimination with psychological well-being and the moderating role of interdependent self-construal in the association of perceived discrimination with psychological well-being. According to the results, perceived discrimination was positively related to separation and negatively to integration, but was related neither to independent nor to interdependent self-construal. Perceived discrimination was, also, positively related to depression directly and indirectly. Fewer depressive symptoms were reported by those immigrants who face discrimination but also select separation. Immigrants with high levels of interdependence, also, do seem to be protected from depression and anxiety. The interpretation of these findings signifies that, when immigrants who perceive discrimination choose separation from the host country, they may reduce their depression feelings, by fitting into relationships with in-group members. Interdependence and the perception of immigrants self as a social unit, also, may act protectively for their psychological well-being, enhancing the identification with the in-group, as well.

Discrimination is one of the major stressors that confront ethnic minority and immigrant groups. Even though discrimination is a real and daily experience for many minority groups, it is difficult to determine it objectively (Paradies, 2006). This paper considers only perceived personal discrimination, namely the perception of receiving an unfair and differential treatment on the basis of ethnicity or race (Blank, Dabady, & Citro, 2004; Schmitt, Branscombe, Posmes, & Garcia, 2014).

Theoretically, the present study is based on the rejection-identification model (Branscombe, Schmitt, & Harvey, 1999; Cronin, Levin, Branscombe, van Laar, & Tropp, 2011; Postmes & Branscombe, 2002) that derives from social identity theory. Social identity theory (Tajfel & Turner, 1986) suggests that individuals strive to maintain a positive social identity being formed mainly from comparisons between in-groups and out-groups, in which in-group favouritism prevails, boosting self-esteem. Social groups who face discrimination maintain psychological well-being by becoming more identified with their own ethnic in-group and rejecting the out-group members and their negative evaluations (Branscombe et al., 1999; Cronin et al., 2011; Jasinskaja-Lahti, Liebkind, & Solheim, 2009; Postmes & Branscombe, 2002).

## Perceived Discrimination and Psychological Well-Being

Several studies have demonstrated that perceived discrimination holds a negative relationship to mental health and physical health outcomes (Gee, Michael-Spencer, Chen, Yip, & Takeuchi, 2007; Paradies, 2006), like more depressive and anxiety symptoms (Schmitt et al., 2014), higher suicide and psychosis rates, and various physical health problems (Chakraborty & Mckenzie, 2002; Franzini & Fernandez-Esquer, 2004; Pascoe & Richman, 2009). Furthermore, this relationship to health is direct and/or indirect (Heim, Hunter, & Jones, 2010; Lindert, Korzilius, Van de Vijver, Kroon, & ArendsToth, 2008).

## The Role of Acculturation Attitudes: Separation as a Mediator

There is evidence that perceived discrimination is related to acculturation process, namely to all those changes that arise when individuals and groups of different backgrounds come into contact (Sam, 2006). Berry (1997) proposed that individuals may adopt different attitudes in their effort to maintain their cultural identity and participate in the larger society. Thus, *integration* may facilitate maintenance of fundamental cultural traits, and, at the same time, enables the individual to participate in the wider social network. *Assimilation* refers to a person’s lack of interest in maintaining his/her own cultural identity while focusing on daily interaction with other cultures. *Separation* is defined as the immigrants’ tendency to remain separate from the host country and to focus on the culture of origin. Finally, *marginalization* occurs when there is little interest in both cultural maintenance and contact and participation.

Longitudinal evidence (Ramos, Cassidy, Reicher, & Haslam, 2016), as well as a meta-analytic review by Schmitt et al. (2014), suggest that perceived discrimination is a negative experience predisposing immigrants towards the pursue of particular acculturation attitudes. Several studies suggest that individuals experiencing high discrimination may seek separation from the mainstream community (Berry, 2001; Berry, Phinney, Sam, & Vedder, 2006). In contrast, when individuals perceive low discrimination, they are more likely to endorse either integration or assimilation (Jasinskaja-Lahti, Liebkind, Horenczyk, & Schmitz, 2003; Sam & Berry, 2010).

A number of studies indicate that integration is preferred by most immigrants as it can promote well-being and enhances adaptation (Berry, 1997; Berry et al., 2006; Papastylianou, 1992, 2000). However, immigrants who pursue separation seem to report more in-group contact and connectedness with their ethnic community and better psychological well-being (Gungor, 2007; Jasinskaja-Lahti et al., 2003; Musso, Inguglia, & Coco, 2015; Safdar, Calvez, & Lewis, 2012; Safdar, Lay, & Struthers, 2003). The mediating role of separation in the relationship of perceived discrimination to psychological well-being was not examined so far. However, other research findings suggest that the direct negative role of perceived discrimination to well-being could be suppressed by the positive effects of coping via ethnic identification and identification with in-group (Awad, 2010; Bergman, Horenczyk, & Abramovsky-Zitte, 2017; Branscombe et al., 1999; Cronin et al., 2011; Schmitt & Branscombe, 2002).

## The Role of Self-Construals: Interdependent Self-Construal as a Moderator

The term self-construal describes how individuals from different cultural backgrounds define and make meaning of the self (Kim, Triandis, Kagitcibasi, Choi, & Yoon, 1994; Markus & Kitayama, 1991; Triandis, 2001). *Independent* self-construal has been used to refer to individuals who perceive themselves to be separate from others, and having as an aim the accomplishment of personal goals (Cross, Hardin, & Gercek-Swing, 2011; Markus & Kitayama, 1991; Singelis, 1994). In contrast, *interdependent* self-construal refers to individuals who perceive themselves to be defined by their relations to others, and having as an aim the acceptance by the in-group and the maintenance of harmony with significant others (Cross & Madson, 1997; Markus & Kitayama, 1991; Singelis, 1994).

Immigrants with different self-construals tend to differ in their acculturation attitudes (Liem, Lim, & Liem, 2000; Yamada & Singelis, 1999). While integration is related to equally advanced independence and interdependence (Yamada & Singelis, 1999), research findings relate separation to interdependent self-construal and assimilation to independent self-construal (Liem et al., 2000). Moreover, people with interdependent self-construal tend to keep a distance from out-groups (Triandis, 1994), to maintain their culture of origin and perceive more discrimination (Hunter, 2008). However, because they are more committed to their heritage culture group, they avoid rejection from their own ethnic in-group and report better psychological well-being (Branscombe et al., 1999; Ferenczi, Marshall, & Bejanyan, 2015; Gungor, 2007; Phalet & Hagendoorn, 1996).

## The Present Study: The Research Context and Hypotheses

### The Research Context

In recent decades, an increasing number of people have immigrated to Greece from the Balkans and several Asian countries (Κuriakou, 2004). The present study included participants from two immigrant populations in Greece, Albanians and Indians. Regarding the Albanian immigrant community, it represents the largest immigrant community in Greece, while Indian community, on the other hand, counts more than 10,000–15,000 (Μarkoutsoglou, Kassou, Mosxobos, & Ptoxos, 2006).

Most studies in Greece suggest that Albanian immigrants in Greece prefer integration or assimilation (Georgas & Papastulianou, 1993; Motti-Stefanidi, Dalla, Papathanasiou, Takis, & Pavlopoulos, 2006; Papastylianou, 1992, 2000), while immigrants coming from more culturally distant countries, like India, report separation (Besevegis & Pavlopoulos, 2008; Kateri, Tsouvelas, & Karademas, 2019; Tsouvelas & Pavlopoulos, 2018). Immigrants in Greece are generally perceived as a threat to Greek cultural identity, social security and welfare systems (Triantafyllidou, 2000). However, high group discrimination in Albanian and Pontian immigrants in Greece was not necessarily resulted in high personal discrimination if protective factors were present (e.g., high self-esteem, and fewer stressors in immigrants’ lives) (Motti-Stefanidi & Asendorpf, 2012). Social support was, also, associated positively with separation of Indian immigrants in Greece minimizing the levels of immigrant’s depression who choose separation (Kateri et al., 2019).

### Hypotheses

Based on the rejection-identification model (Branscombe et al., 1999; Cronin et al., 2011; Postmes & Branscombe, 2002), the acculturation attitude of separation and interdependent self-construal were examined as factors directing immigrants to their in-group, minimizing the negative effects of perceived discrimination to their psychological well-being. Possible differences between Albanians and Indians in mediation and moderation analyses were, also, examined, given that these two groups are different regarding cultural traits, acculturation and psychological well-being (Besevegis & Pavlopoulos, 2008; Μarkoutsoglou et al., 2006).

(H1) It was hypothesized a positive relationship of perceived discrimination to separation and a negative relationship of perceived discrimination to integration and assimilation.

(H2) It was hypothesized a positive relationship of perceived discrimination to interdependent self-construal and a negative relationship of perceived discrimination to independent self-construal.

(H3) It was hypothesized a direct relationship of perceived discrimination to depression and anxiety.

(H4) It was hypothesized that the indirect effects of perceived discrimination on psychological well-being would be through the acculturation attitude of separation. Moreover, a positive relationship of perceived discrimination to separation was expected to result in turn in less anxiety and depression.

(H5) It was hypothesized that the strength of the relationship between perceived discrimination and anxiety/depression would depend on (or be moderated by) the level of interdependent self-construal. That is, at the higher levels of interdependent self-construal immigrants would report relatively lower levels of anxiety and depressive symptoms.

## Method

### Measures

Factor analyses were conducted for all measures; analyses were carried out separately for Indians and Albanians. Factorial agreement between the two samples was addressed next, by using Tucker’s Phi, coefficient of congruence. According to Van de Vijver and Leung (1997), values higher than 0.090 indicate factorial similarity.

#### Perceived Discrimination Scale

The Perceived Discrimination Scale was developed by Phinney, Madden, and Santos (1998). Respondents reported to what extent they agreed or disagreed with statements about differential and negative treatment because of their ethnicity using a scale ranging from “never” (1) to “very often” (5). Given that the scale has not been evaluated in a Greek context, a confirmatory factor analysis (CFA), using Lisrel 8.30 (Joresjog & Sorbom, 1996), was conducted. The analysis confirmed the existence of a single general factor (χ^2^ = 17.53, *df* = 14, RMSEA = 0.03, AGFI = 0.95, NNFI = 0.99, CFI ≥ 0.99, *p* < .05). Coefficient of congruence (Tucker’s phi) was 0.99. In the current study, Cronbach’s alpha was 0.86.

#### Acculturation Attitudes Scale

This scale was developed for purposes of the international study conducted by Berry et al. (2006). It consists of 20 items and measures four factors, namely, assimilation, integration, separation and marginalization. The items refer to five domains of life, including marriage, language, cultural traditions, social activities and friends. Participants responded to what extent they agreed or disagreed with each statement, using a Likert-type scale ranging from “strongly disagree” (1) to “strongly agree” (5). In the present study, CFA conducted for this scale, given that it was the first time that the scale was used in Greek. The four–factor model moderately fit the data (χ^2^ = 228.56, *df* = 144, RMSEA = 0.05, AGFI = 0.90, NNFI = 0.86, CFI = 0.90, *p* < .001). Coefficients of congruence (Tucker’s phi) were 0.98 for assimilation, integration and separation, and 0.93 for marginalization (5). Cronbach’s alpha was 0.60 for integration, 0.71 for separation, 0.61 for marginalization and 0.57 for assimilation.

#### Self-Construal Scale

The Self-Construal Scale (SCS) is a 30-item questionnaire that assesses both independent and interdependent self-construal (Singelis, 1994). Each dimension is measured with 15 items. Participants responded to what extent they agreed or disagreed with each statement, using a Likert-type scale ranging from 1 (strongly disagree) to 5 (strongly agree). This scale was used in this study as adapted in Greek by Georgas, Berry, Van de Vijver, Kagitcibasi, and Poortinga (2006). Coefficients of congruence (Tucker’s phi) was 0.92 for interdependent and 0.80 for independent self-construal. Cronbach’s alpha was 0.62 for the independent self-construal and 0.63 for the interdependent self-construal.

#### Center for Epidemiological Studies Depression Scale (CES-D)

Depression was measured with the CES-D scale (Radloff, 1977) as adapted in Greek by Fountoulakis et al. (2001). It consists of 20 items and participants responded to what extent they agreed or disagreed with each statement, using a Likert-type scale ranging from 1 (strongly disagree) to 5 (strongly agree). Coefficient of congruence (Tucker’s phi) was 0.81. Cronbach’s alpha was 0.83.

#### State Anxiety Inventory

Anxiety was measured with the State Anxiety Inventory (Spielberger, Gorush, & Lushene, 1970) as adapted in Greek by Liakos and Giannitsi (1984), which evaluates the anxiety the person feels at the moment of measurement. This scale consists of 20 items and participants responded to what extent they agreed or disagreed with each statement, using a Likert-type scale ranging from 1 (not at all) to 4 (very much). Coefficient of congruence (Tucker’s phi) was 0.55. Cronbach’s alpha was 0.85.

### Procedure

The inclusion criterion for participants in the study was being first generation and older than 18 years old, due to various adaptation differences in these groups (Berry et al., 2006; Sam, 2006). Immigrants were recruited mainly in their work place using a convenience sampling technique. Completion of the questionnaire took place during their break and participation in the study was voluntary. Each measure was translated to Punjabi and Albanian from English and translated back to English (Brislin, 1970). The Indian questionnaire was translated into Punjabi by the Department of the Linguistic Studies of the Punjabi University in India and back translated into English by an Indian living in Greece for more than 10 years, with a MSc. in Sociology from a Greek university. The translation into Albanian was performed by an Albanian living in Greece for more than 20 years, with a BSc. degree of Sociology from a Greek university and back translated into English by a second generation Albanian immigrant who was born in Greece and was a student at School of Education. In order to examine the indirect effects of perceived discrimination on anxiety and depression through acculturation attitudes as well as the moderation role of self-construal, a set of analyses were performed in PROCESS, which is a freely-available computational tool for SPSS and SAS, developed by Hayes (2013).

### Participants

Two hundred and thirty-three immigrants in Greece participated in the study. One hundred and fifteen were Indians and one hundred and eighteen were Albanian. In a preliminary analysis, Albanians and Indians were examined regarding their demographic characteristics. Most of the participants were male (75.4% of Indians and 56.4% of the Albanian sample), married (77.1% of Indians and 78.6% of Albanians) and unskilled workers (62.2% of Indians and 57.3% of Albanians). The average age was 33.0 years for Indians (*SD* = 8.0) and 35.0 years for Albanians (*SD* = 8.0), while the age range for both Albanians and Indians was 18–64. Their average years of education was 11.2 years (*SD* = 2.7) for Indians and 11.8 years (*SD* = 2.4) for Albanians. Regarding the years of residence in Greece, the average years were 7.7 (*SD* = 3.9) for Indians and 10.7 (*SD* = 4.2) for Albanians. As far as their religion was concerned, the majority of Indians were Sikhs (81.3%), while heterogeneity was noticed in the Albanian sample: 31.2% were Orthodox’s, 31.2% were Muslims and 21.4% were Atheists.

Also, in preliminary analyses, Albanian and Indian immigrants were compared regarding years of residence in Greece, age, years of education, anxiety and depression. Albanian immigrants had lived for in Greece for longer compared to Indians and this difference was statistically significant, *t*(219) = −5.38*, p < .*001. The differences between the two ethnic groups were not significant as far as age, *t*(223) = −1.23, *p > .*05, and years of education were concerned, *t*(214) = −1.58*, p > .*05. *t*-test indicated a significant difference, regarding anxiety, *t*(156) = −3.63, *p* < .001, and depression, *t*(187) = −2.12, *p* < .05, regarding ethnicity as well. For this reason, the mediation analyses were, also, performed separately for Albanians and Indians and moderation analyses were first performed without covariates and then with ethnicity as one.

## Results

Table 1 presents the bivariate correlations among perceived discrimination, psychological well-being, acculturation attitudes, and independent/interdependent self-construal. According to the results, perceived discrimination was positively related to separation (*r =* .46*, p <* .01) and negatively to integration (*r =* −.39, *p <* .01). It was not related to assimilation (*r* = −.02, *p* > .05) and marginalization (*r* = .03, *p* > .05) *(H1).* Regarding self-construal, perceived discrimination was not related to independent (*r* = −.09, *p* > .05) or interdependent self-construal (*r* = −.10, *p* > .05) *(H2).*

**Table 1:** Means, Standard Deviations (SD) and Bivariate Correlations Among Psychological Well-Being, Acculturation Attitudes, Perceived Discrimination, Independent and Interdependent Self-Construal

Study Variables	*M*	*SD*	1	2	3	4	5	6	7
**1.** Anxiety	44.50	10.74	1.00						
**2.** Depression	17.85	10.22	.63**	1.00					
**3.** Integration	19.78	3.72	.05	−.05	1.00				
**4.** Separation	14.50	5.47	−.02	−.14	−.36**	1.00			
**5.** Assimilation	9.02	3.72	−.03	.15	−.03	.13	1.00		
**6.** Marginalization	8.49	3.60	.04	.14	−.18*	.30**	.42**	1.00	
**7.**Perceived Discrimination	11.99	7.82	.10	.06	−.39**	.46**	−.02	.03	1.00
**8.** Independent Self-Construal	4.98	.66	−.10	.00	.21**	−.05	.09	−.03	−.09
**9.** Interdependent Self-Construal	5.08	.63	−.06	−.07	.20**	−.16*	−.03	−.08	−.10

*Note*. *N* = 233.

*p < .05. **p < .01.

**Figure 1:**
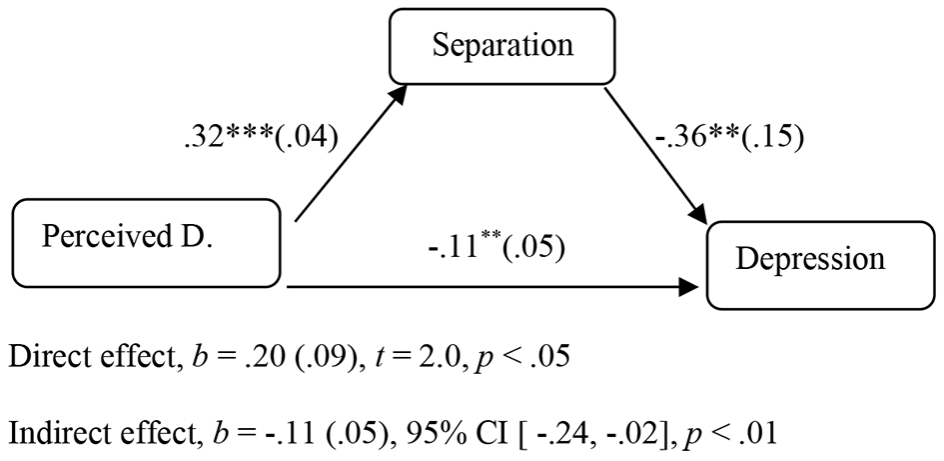
Graphical Representation of the Statistically Significant Indirect (Mediation) Effects (*B*-Coefficients, *SE* in Parenthesis) *Note*. Perceived D. = Perceived Discrimination. *p < .05. **p < .01. ***p < .001.

Regarding the direct effects of perceived discrimination on depression and anxiety *(H3),* the results indicated a statistically significant direct effect of perceived discrimination on depression, *B* = 0.20, *SE* = 0.09, *p* < .05, 95% CI [0.01, 0.40]. Regarding the indirect effects of perceived discrimination on depression and anxiety (*H4*), it was examined whether separation, integration, assimilation, and marginalization acted as mediators. The indirect effect of perceived discrimination on depression through separation was statistically significant, *B* = −0.11, *SE* = 0.05, *p* < .01, 95% CI [−0.24, −0.02] (see Figure 1, Table 2). However, the indirect effect of perceived discrimination on anxiety through separation was not statistically significant, *B* = −0.05, *SE* = 0.07, *p* > .05, 95% CI [−0.21, 0.08]. No indirect effects on the relationship between perceived discrimination and psychological well-being were found for integration, assimilation, and marginalization (see Table 2). Next, it was examined whether the indirect role of separation to the relationship between perceived discrimination and depression depend on ethnicity (i.e., Albanians vs. Indians). Analyses revealed that the indirect role of separation was statistically significant for both Albanians, *B* = −0.49, *SE* = −0.06, 95% CI [−0.15, −0.00], and Indians, *B* = 0.50, *SE* = −0.08, *p* < .01, 95% CI [−0.22, −0.00].

**Table 2:** The Indirect and Direct Effects of Perceived Discrimination on Depression and Anxiety Through Acculturation Attitudes (Mediation)

Bootstrapping 95% CIs^a^										
Acculturation Attitudes	*B*	*SE*	*p*	Indirect Effects		*B*	*SE*	*p*	Direct Effects	
				*LL*	*UL*				*LL*	*UL*
Dependent Variable: Depression										
Integration	0.00	0.05	.95	−0.11	0.09	0.04	0.10	.76	−0.17	0.25
Assimilation	−0.00	0.01	.28	−0.03	0.02	0.01	0.10	.88	−0.18	0.21
Separation	−0.11	0.05	.01	−0.24	−0.02	0.20	0.09	.03	0.01	0.40
Marginalization	0.00	0.01	.06	−0.02	0.04	0.05	0.10	.61	−0.15	0.25
Dependent Variable: Anxiety										
Integration	−0.06	0.05	.15	−0.20	0.01	0.20	0.12	.10	−0.04	0.46
Assimilation	0.00	0.01	.44	−0.01	0.07	0.06	0.12	.60	−0.17	0.30
Separation	−0.05	0.07	.46	−0.21	0.08	0.20	0.12	.11	−0.05	0.46
Marginalization	−0.00	0.01	.65	−0.01	0.04	0.12	0.11	.29	−0.10	0.34

^a^Bootstrapping bias corrected and accelerated (5,000 bootstrap samples). Indirect and direct effects are significant at *p* < .05 for the 95% bootstrap confidence intervals, when the derived intervals do not include values of zero.

**Figure 2:**
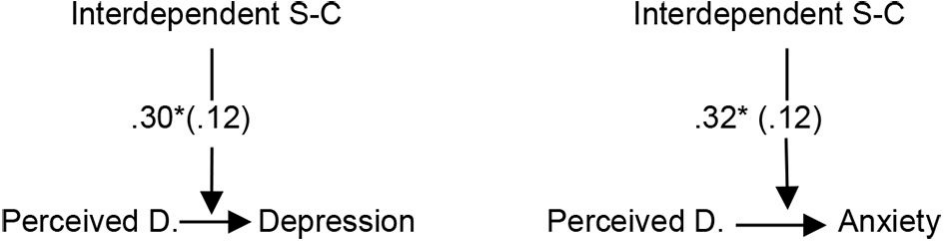
Graphical Representation of the Statistically Significant Moderation Effects (*B*-Coefficients, *SE* in Parenthesis) *Note*. Perceived D. = Perceived Discrimination; S-C = Self-Construal. *p < .05.

**Figure 3:**
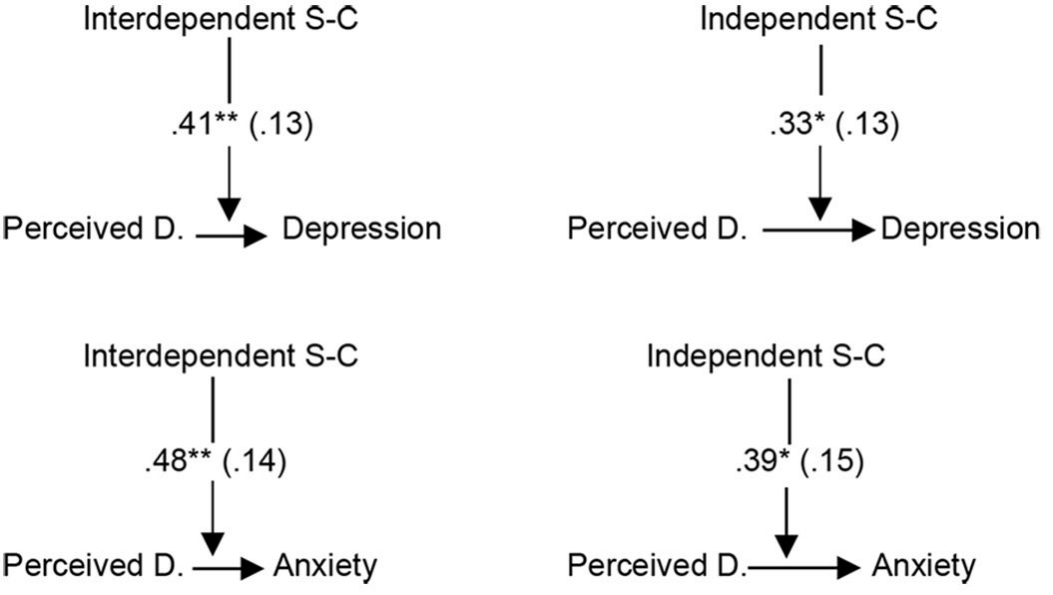
Graphical Representation of the Statistically Significant Moderation Effects Using Ethnicity as Covariate (*B*-Coefficients, *SE* in Parenthesis) *Note*. Perceived D. = Perceived Discrimination; S-C = Self-Construal. *p < .05. **p < .01.

Subsequently, it was tested whether interdependent self-construal moderate the relationship of perceived discrimination to depression and anxiety *(H5).* The variables were mean-centered first (Hayes, 2013). As shown in Table 3, the correlation between perceived discrimination and depression, as well as between perceived discrimination and anxiety was statistically significant only at the higher levels of interdependent self-construal (+1 *SD*). No moderation effect of independent self-construal was found. The statistically significant moderation effects of interdependent self-construal on the relationship of perceived discrimination to anxiety and depression are presented in Figure 2. When ethnicity was used as a covariate for the moderation analyses, the relationship between perceived discrimination and depression was statistically significant at the medium level of independent self-construal, *B* = 0.33, *SE* = 0.13, *p* < .05, 95% CI [0.06, 0.61] and at the higher level of interdependent self-construal, *B* = 0.41, *SE* = 0.13, *p* < .01, 95% CI [0.15, 0.67]. Regarding the relationship between perceived discrimination and anxiety, it was statistically significant at the higher level of interdependent, *B* = 0.48, *SE* = 0.14, *p* < .01, 95% CI [0.21, 0.76] and independent self-construal, *B* = 0.39, *SE* = 0.15, *p* < .05, 95% CI [0.09, 0.69]. The statistical significant moderation effects using ethnicity as covariate are presented in Figure 3. According to this figure, both independent and interdependent self-construal moderated the relationship of perceived discrimination to anxiety and depression.

**Table 3:** Bootstrapping Results for Moderation Effects of Interdependent/Independent Self-Construal on the Relationship of Perceived Discrimination to Depression and Anxiety

Self-Construal	*B*	*SE*	*t*	*p*	95% CI^a^	
					*LL*	*UL*
Dependent Variable: Depression *t*						
Interdependent Self-Construal						
Low (−1 *SD*)	−0.21	0.12	−1.7	.08	−0.45	0.02
*M*	0.04	0.09	0.51	.60	−0.13	0.23
High (+1 *SD*)	0.30*	0.12*	2.3	.01	0.05	0.56
Independent Self-Construal						
Low (−1 *SD*)	−0.12	0.12	−0.96	.33	−0.36	0.12
*M*	0.05	0.09	0.61	.53	−0.12	0.24
High (+1 *SD*)	0.23	0.13	1.76	.07	−0.02	0.50
Dependent variable: Anxiety						
Interdependent Self-Construal						
Low (−1 *SD*)	−0.12	0.18	−0.66	.50	−0.48	0.23
*M*	0.10	0.11	0.92	.35	−0.11	0.32
High (+1 *SD*)	0.32*	0.12*	2.62	.04	0.08	0.57
Independent Self-Construal						
Low (−1 *SD*)	−0.01	0.16	−0.06	.95	−0.34	0.32
*M*	0.11	0.11	0.97	.33	−0.11	0.33
High (+1 *SD*)	0.23	0.14	1.64	.10	−0.04	0.51

*Note*. *SD* = standard deviation; CI = confidence intervals; *SE* = standard error; *LL* = lower limit; *UL* = upper limit.

^a^Bootstrapping bias corrected and accelerated (5,000 bootstrap samples). Intervals that do not contain zero are deemed to be significant.

*p < .05.

## Discussion

The results of the present study in Albanian and Indian immigrants in Greece supported rejection-identification model (Branscombe et al., 1999; Cronin et al., 2011; Postmes & Branscombe, 2002). Separation mediated the relationship of perceived discrimination to depression, reducing depressive symptoms, and, at the higher levels of interdependent self-construal immigrants reported relatively lower levels of anxiety and depressive symptoms.

Initially, in accordance to the study hypothesis, separation was positively related to perceived discrimination, whereas integration was related negatively (Jasinskaja-Lahti et al., 2003). Interestingly assimilation was not connected to perceived discrimination at all. A possible explanation might be that assimilation promotes personal goals, acquisition of social skills, language proficiency and sustained contact with host community members (Ataca & Berry, 2002; Kosic, Mannetti, & Sam, 2006). It is possible that perceived discrimination connects to assimilation indirectly, through factors acting as mediators, like acceptance by hosts (Motti-Stefanidi, Pavlopoulos, & Asendorpf, 2018).

Secondly, the relationship of perceived discrimination to self-construal was examined. Contrary to the study hypothesis, no statistical significant relationship of these variables was identified. In the context of the present study, perceived discrimination can be conceived as an ego-threat condition that may elicit interpersonal behaviours depending on the self-esteem of the individuals. In the case of independence, perceived discrimination of high self-esteem individuals may elicit self-serving bias, while, in the case of interdependence, perceived discrimination of low self-esteem individuals may elicit interpersonal appraisals (Vohs & Heatherton, 2001). Future study of the role of self-esteem in the relationship of perceived discrimination to self-construal is needed to test these assumptions.

Third, it was examined the relationship of perceived discrimination to anxiety and depression. In accordance with the study hypothesis, a direct relationship of perceived discrimination to depression was identified, confirming previous studies which suggested that perceived discrimination is a negative factor regarding immigrants’ psychological well-being (Gee et al., 2007; Paradies, 2006; Pascoe & Richman, 2009).

Regarding the indirect relationship of perceived discrimination to psychological well-being, separation was found to operate as a mediator. Fewer depressive symptoms were reported by those immigrants who face discrimination but also select separation. The experience of discrimination is conceptualized as a threat against personal identity and intensifies identification with the in-group (Jetten, Branscombe, Schmitt, & Spears, 2001). Given that separation signifies maintenance of the culture of origin (Sam & Berry, 2010), it may enhance identification with the in-group (Jasinskaja-Lahti et al., 2003). Furthermore, the avoidance of contact with host community members, as another defining feature of separation (Sam & Berry, 2010), may help immigrants feel safer from their rejection (Mossakowski, 2003), by asking and receiving more social support by family and friends (Kateri et al., 2019).

However, it should be noted that, contrary to the study hypothesis (H3), no direct relationship of perceived discrimination to anxiety was found, nor separation was found to operate as a mediator. It is important to note that anxiety and depression are distinct from each other (Crockett, Iturbide, Torres, & Carlo, 2007). Anxiety may be related to factors that were not measured in the present study, such as unemployment, difficulties in language, or lacking official documents or residence permits. It is possible that stress coping mechanisms (Essau & Trommsdorff, 1996) may affect anxiety more than perceived discrimination.

Regarding the role of interdependent self-construal in the relationship between perceived discrimination and depression/anxiety, the results support the moderation hypothesis. Immigrants with high levels of interdependence do seem to be protected from depression and anxiety. It is plausible that the more the devalued groups recognize the prejudice against them, the more they increase their emotional investment in their in-group (where they feel accepted), protecting their psychological well-being (Branscombe et al., 1999; Cronin et al., 2011; Ferenczi et al., 2015).

The differences in the results when ethnicity was included in the analyses seem to be quite interesting. The indirect role of separation to the relationship between perceived discrimination and depression remained statistically significant for both Albanian and Indian immigrants, indicating that separation may prove helpful in the case of perceived discrimination, regardless of the immigrant’s ethnicity (Gungor, 2007; Jasinskaja-Lahti et al., 2003; Musso et al., 2015). Regarding the moderation effects, both interdependent self-construal and independent self-construal seem to minimize the relationship between perceived discrimination and depression and between perceived discrimination and anxiety. It could thus, be assumed that both forms of construal coexist in the development of self, by satisfying the basic human needs of autonomy and relatedness, which are essential for the psychological well-being of any individual (Kagitcibasi, 2005).

### Limitations and Conclusions

A number of limitations need to be considered in the interpretation of the present findings. First of all, internal consistencies were low to moderate for the scales of acculturation attitudes and self-construal. Moreover, although CFA seemed to confirm the existence of four factors (i.e., integration, assimilation, separation, and marginalization) in the Acculturation Attitudes Scale, the relevant indices are not considered to be satisfactory. Furthermore, coefficients of congruence were low for the scales of depression, anxiety, and independent self-construal, indicating factorial dissimilarity between Albanian and Indians for these scales. All these findings are raising concerns about the cross-cultural validity of constructs in non-Western cultures (Heine, Lehman, Peng, & Greenholtz, 2002). Moreover, a convenient sample was used which also represents only a small sub-set of immigrants in Greece, limiting the ability to generalize the results. Furthermore, given the cross-sectional nature of the study design, the direction of causality assumed in the mediation analyses may not be accurate (Maxwell & Cole’s, 2007).

Nevertheless, the present findings make a unique theoretical contribution to the protective role of interdependent self-construal in immigrants who experience discrimination. Social identity theories (Tajfel & Turner, 1986) stress the importance of inclusion for well-being and highlight the negative role of rejection from important social groups. However, interdependent individuals tend to perceive themselves more as a social unit (e.g., in connection with the social context) than as separate, autonomous and unique individuals (Markus & Kitayama, 1991; Singelis, 1994) and this form of self-contrual may have positive consequences in the case of perceived discrimination. Thus, there is a possibility that more interdependent individuals would avoid contacts with out-group members (Triandis, 1994), exposing themselves to a lesser extent to discriminatory events and experience less stress. Interdependent individuals, also, tend to rely more on the approval of the in-groups for their self-validation (Gungor, 2007), increasing social support behaviours (Morry & Kito, 2009).

We must be cautious with the interpretation of the result findings regarding separation. Separation seems to be a tactic that temporarily might ensure wellbeing to some extend to migrants (Berry, 2006). However, it is possible that separation might, be an obstacle in the case of their sociocultural adaptation (Berry, 1997; Berry et al., 2006). Furthermore, separation in the long-term may end in out-group rejection and segregation (Bourhis, Montaruli, El‐Geledi, Harvey, & Barrette, 2010), making immigrants feel more depressed.
